# Supplementary material for: Metabolomic analysis of the impact of MtrA on carbon metabolism in Streptomyces coelicolor
Source: Microbiol Spectr. 2025 Jun 30;13(8):e00096-25. doi: 10.1128/spectrum.00096-25 (PMC12323332; doi:10.1128/spectrum.00096-25)
Supplement: Supplemental material — Fig. S1 to S21; Tables S1 to S7. [file spectrum.00096-25-s0001.pdf]

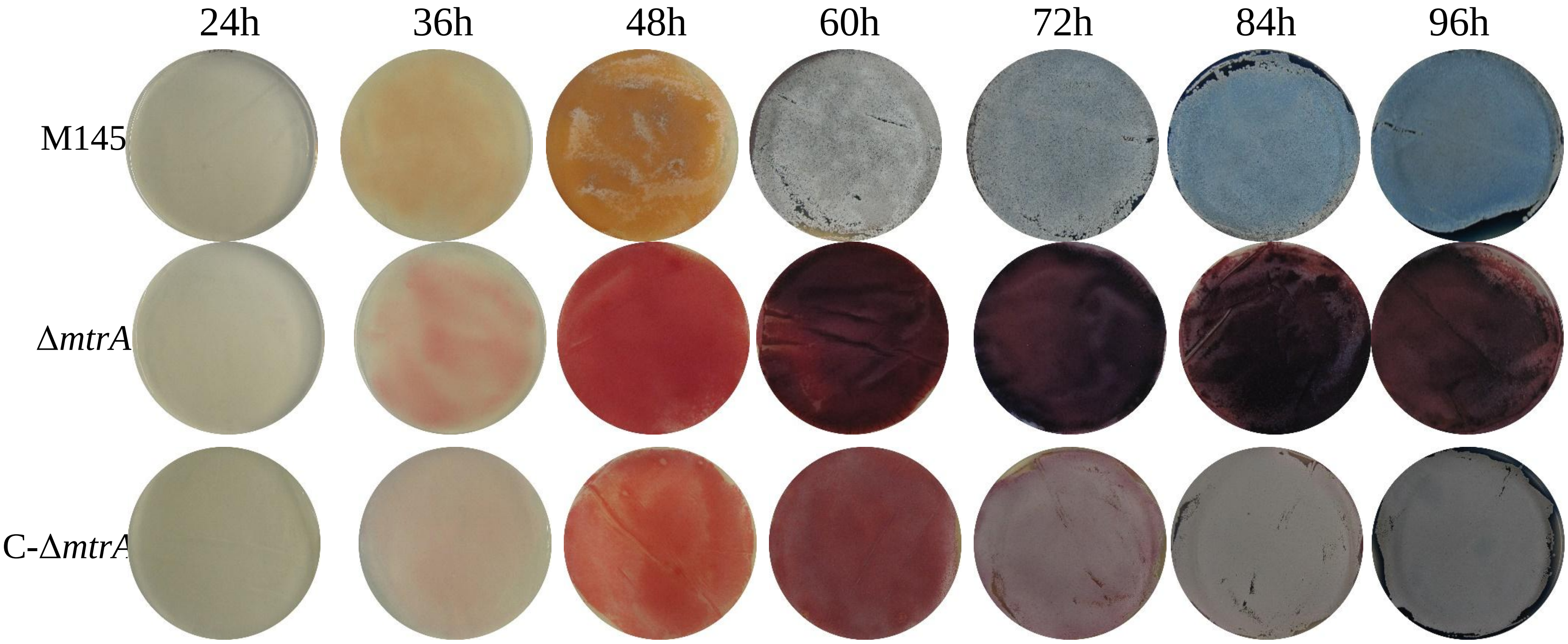

FIG S1 Growth of the wild-type *S. coelicolor* strain M145, Δ*mtrA*, and the complemented strain C-Δ*mtrA* on solid YBP at the indicated times. On YBP, although these strains produced the red (undecylprodigiosin) and blue (actinorhodin) antibiotics, Δ*mtrA* does not form aerial mycelium, thus appearing bald.

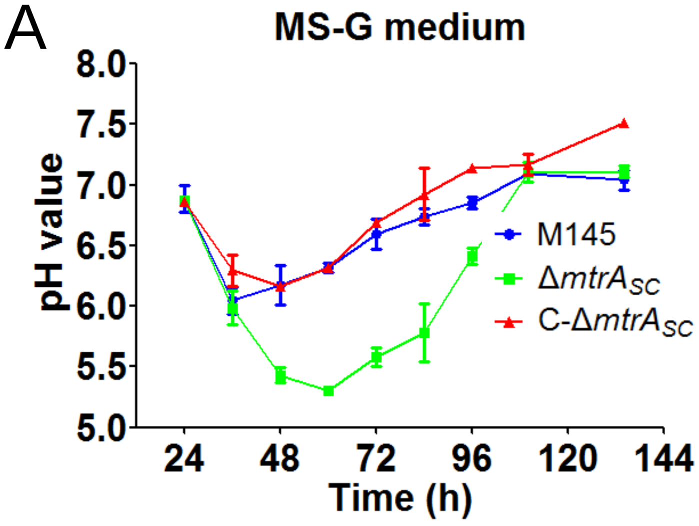

FIG S2 (A) Temporal pH value curve of the wild-type *S. coelicolor* strain M145, Δ*mtrA*, and the complemented strain C-Δ*mtrA* on solid MS. (B) Growth of M145, Δ*mtrA*, and C-Δ*mtrA* on solid MS at the indicated times.

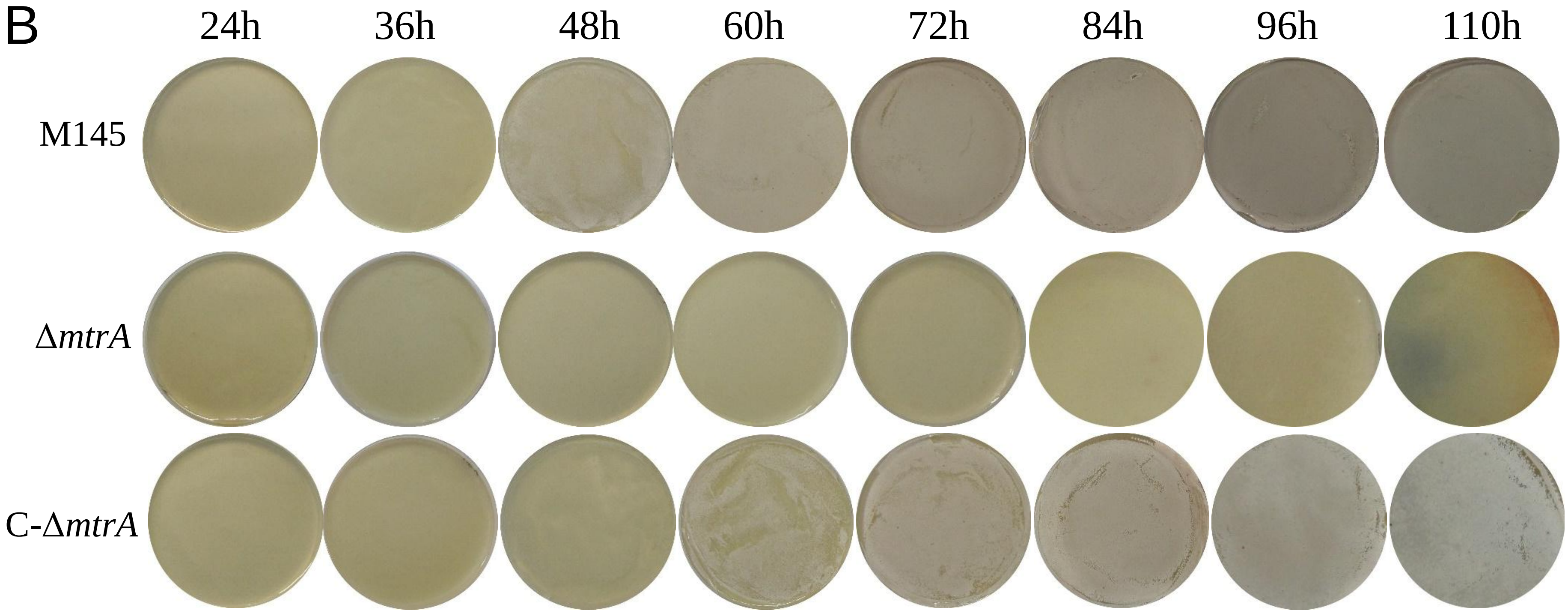

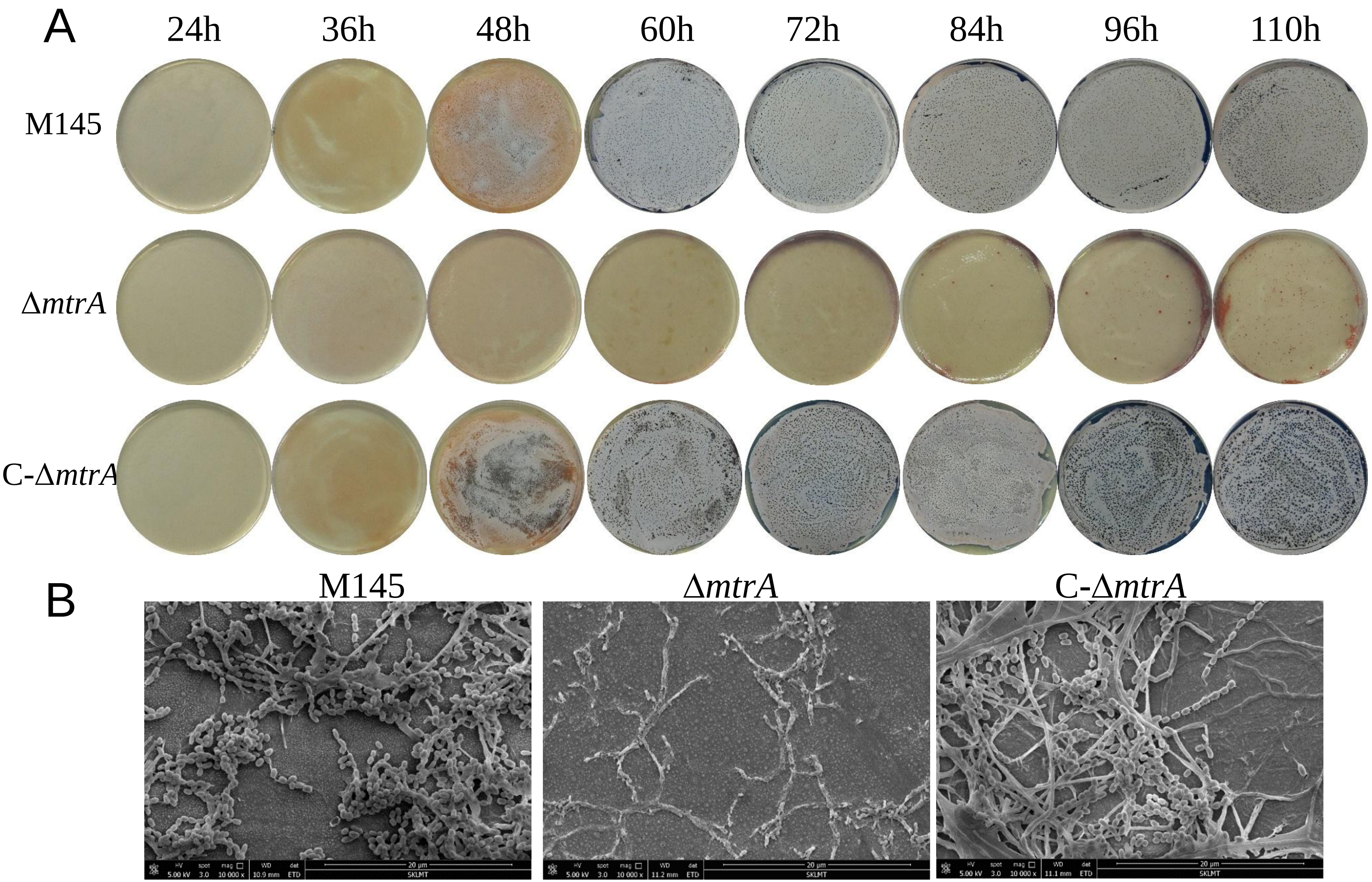

FIG S3 (A) Growth of the wild-type *S. coelicolor* strain M145, Δ*mtrA*, and the complemented strain C-Δ*mtrA* on solid R2YE at the indicated times. Δ*mtrA* does not form aerial mycelium and display a bald phenotype on R2YE. (B) SEM images of M145, Δ*mtrA*, and the complemented strain C-Δ*mtrA* after growth on solid R2YE agar for 5 days. The scale bar is 20 μm.

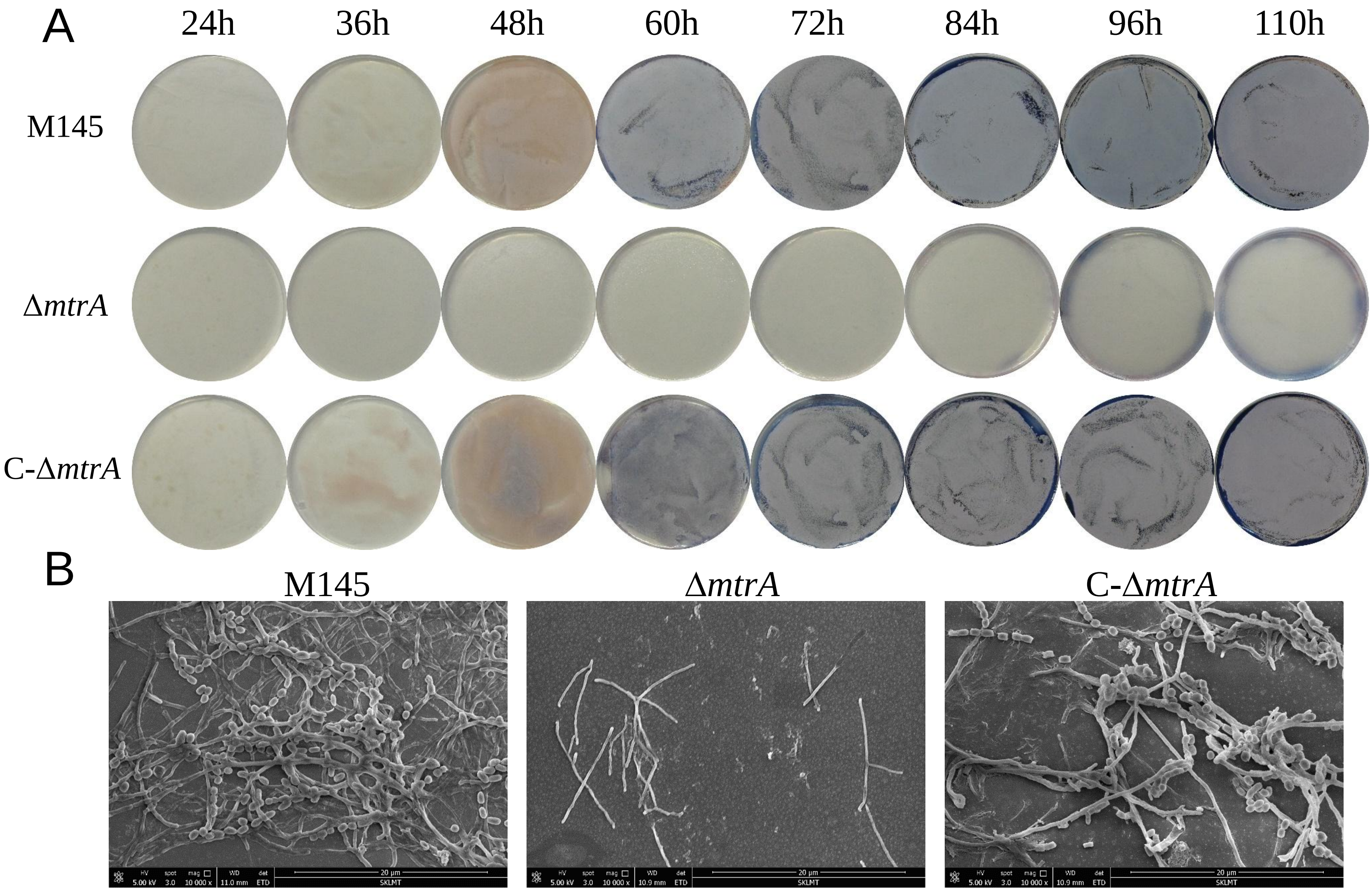

FIG S4 (A) Growth of the wild-type *S. coelicolor* strain M145, Δ*mtrA*, and the complemented strain C-Δ*mtrA* on solid R2 at the indicated times. Δ*mtrA* does not form aerial mycelium and display a bald phenotype on R2. (B) SEM images of M145, Δ*mtrA*, and the complemented strain C-Δ*mtrA* after growth on solid R2 agar for 5 days. The scale bar is 20 μm.

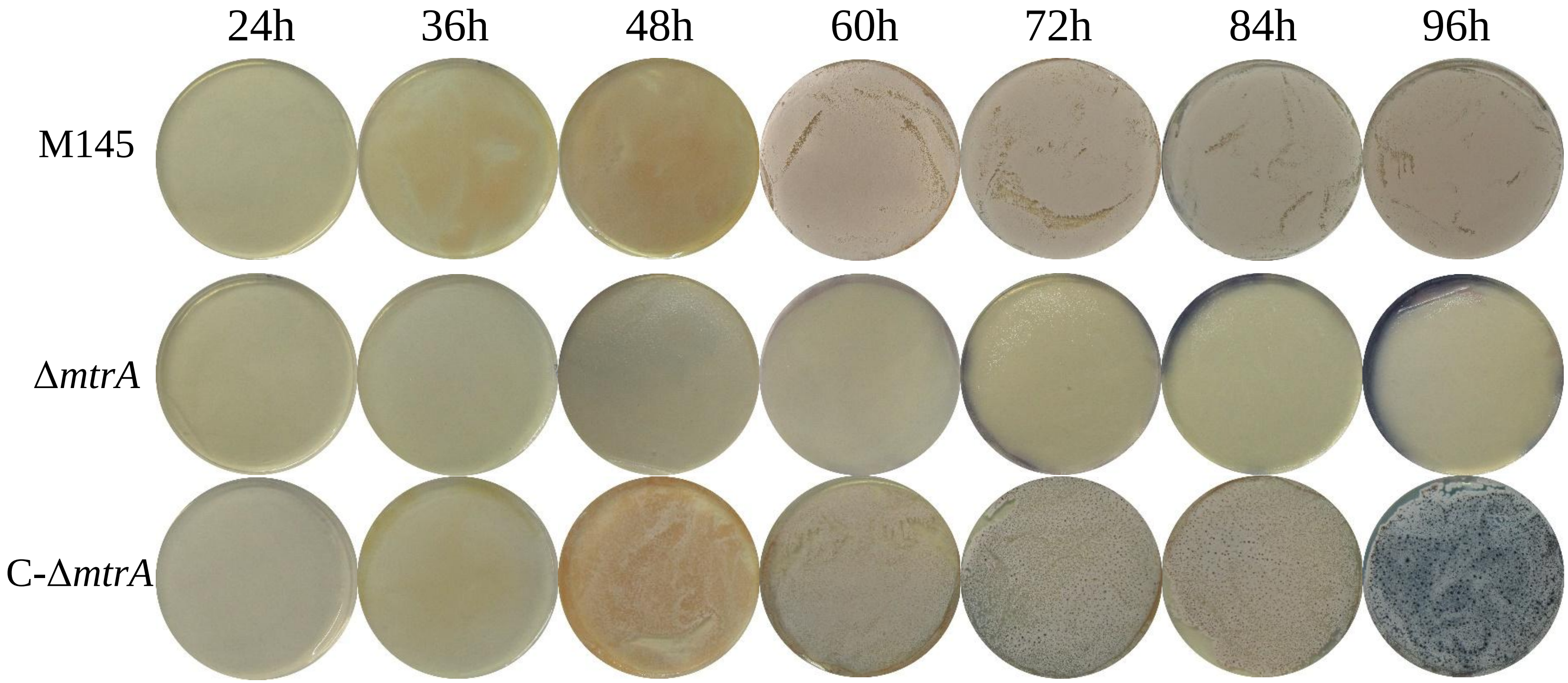

FIG S5 Growth of the wild-type *S. coelicolor* strain M145, Δ*mtrA*, and the complemented strain C-Δ*mtrA* on solid R2 (-proline, +peptone) at the indicated times. Δ*mtrA* does not form aerial mycelium and disply a bald phenotype under this condition.

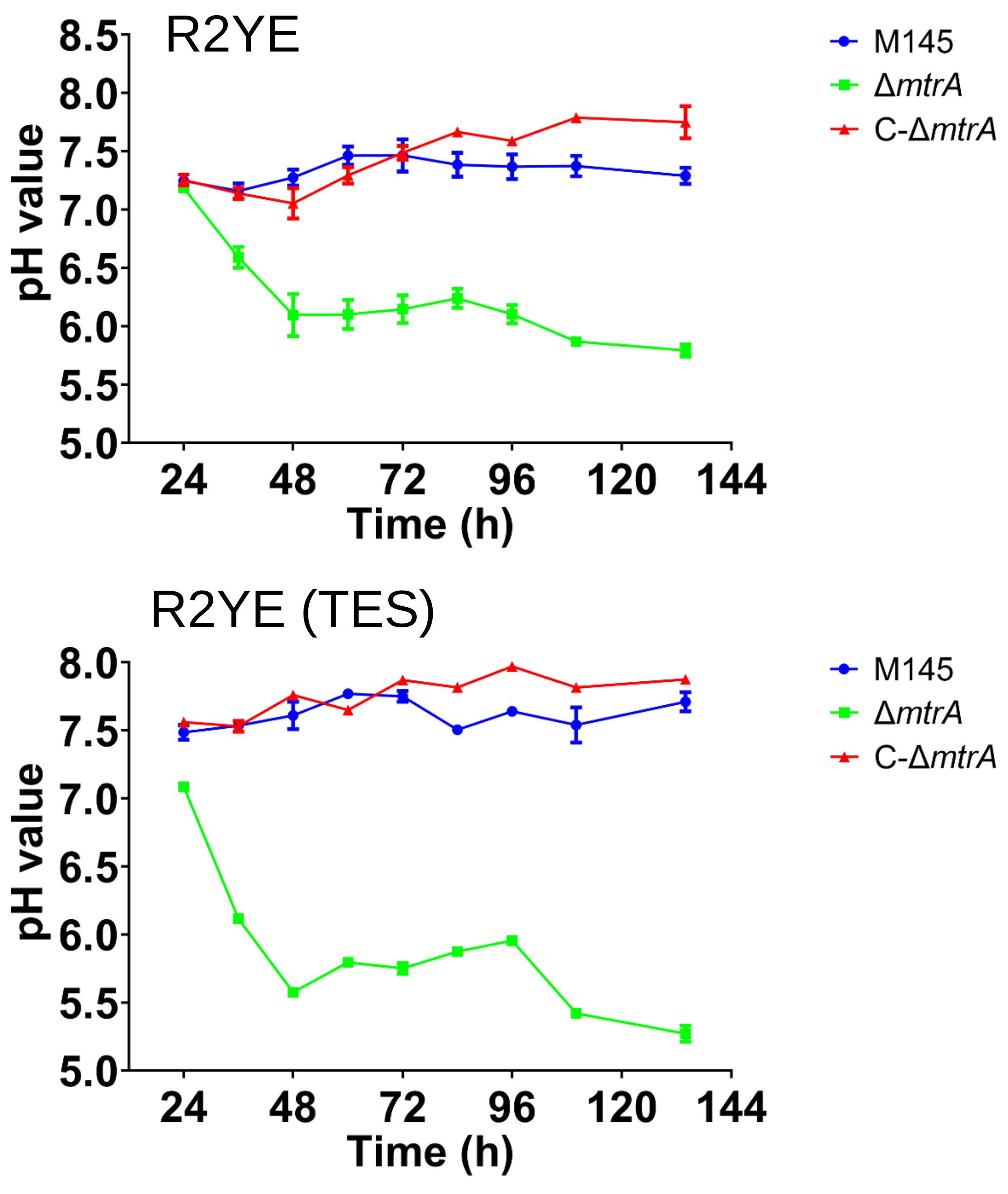

FIG S6 The effect of TES buffer on the pH of growth medium. The pH values of the growth medium were measured for the wild-type strain *S. coelicolor* M145, Δ*mtrA*, and the complemented strain C-Δ*mtrA* grown on R2YE and R2YE lacking TES buffer.

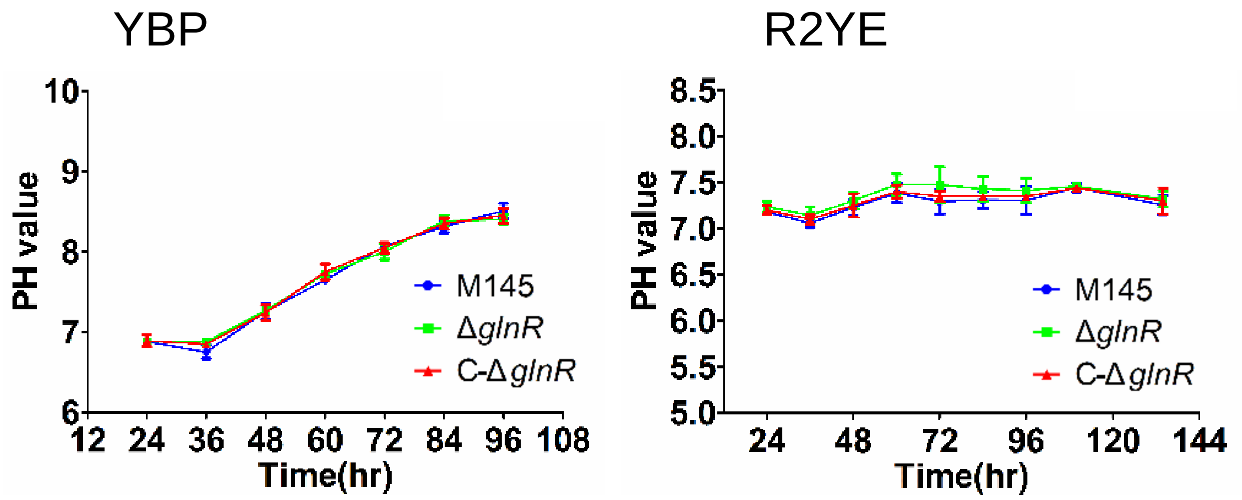

FIG S7 Temporal pH values of the growth medium for *glnR* mutant strain of *S. coelicolor* under different growth conditions. The pH values of the growth medium were measured for M145, the *glnR* deletion mutant Δ*glnR*, and the *glnR*-complemented strain C-Δ*glnR* grown on YBP and R2YE.

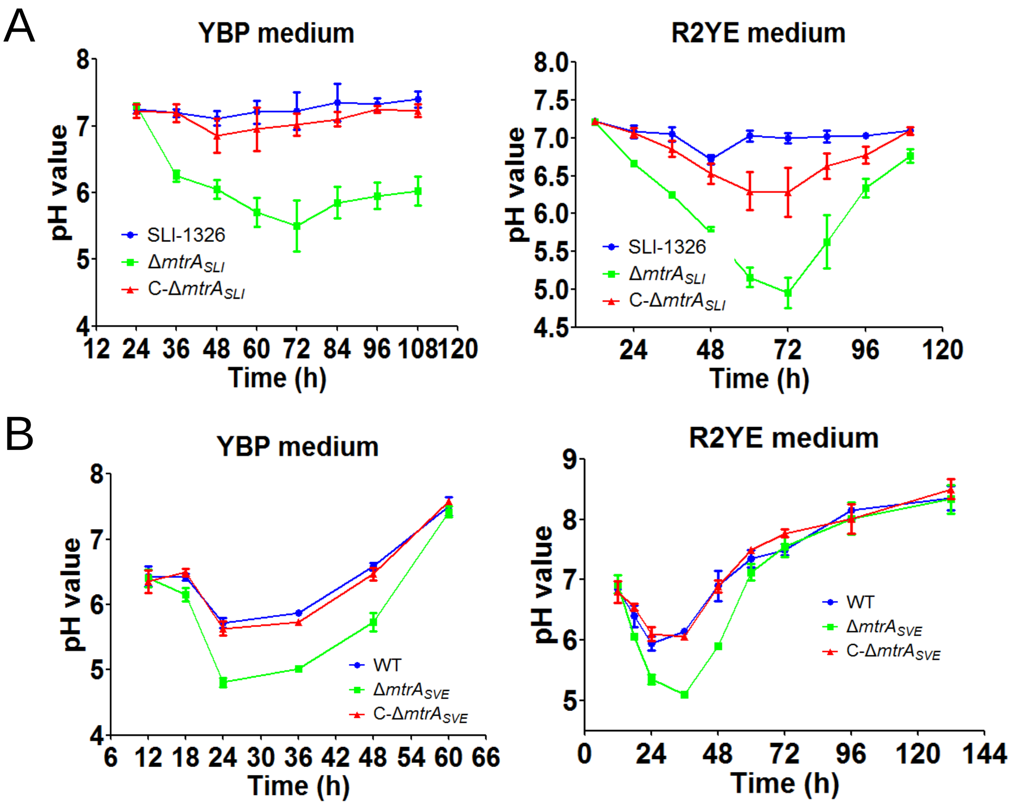

FIG S8 Temporal pH values of the growth medium for *mtrA* mutant strains under different growth conditions. The pH values of the growth medium were measured for (A) the wild-type strain *S. lividans* 1326 (SLI-1326), the *mtrA* deletion mutant of *S. lividans* Δ*mtrA*$_{SLI}$, and the *mtrA*-complemented strain C-Δ*mtrA*$_{SLI}$ grown on YBP and R2YE; and (B) the wild-type strain *S. venezuelae* 10712 (WT), the *mtrA* deletion mutant of *S. venezuelae* Δ*mtrA*$_{SVE}$, and the *mtrA*-complemented strain C-Δ*mtrA*$_{SVE}$ grown on YBP and R2YE.

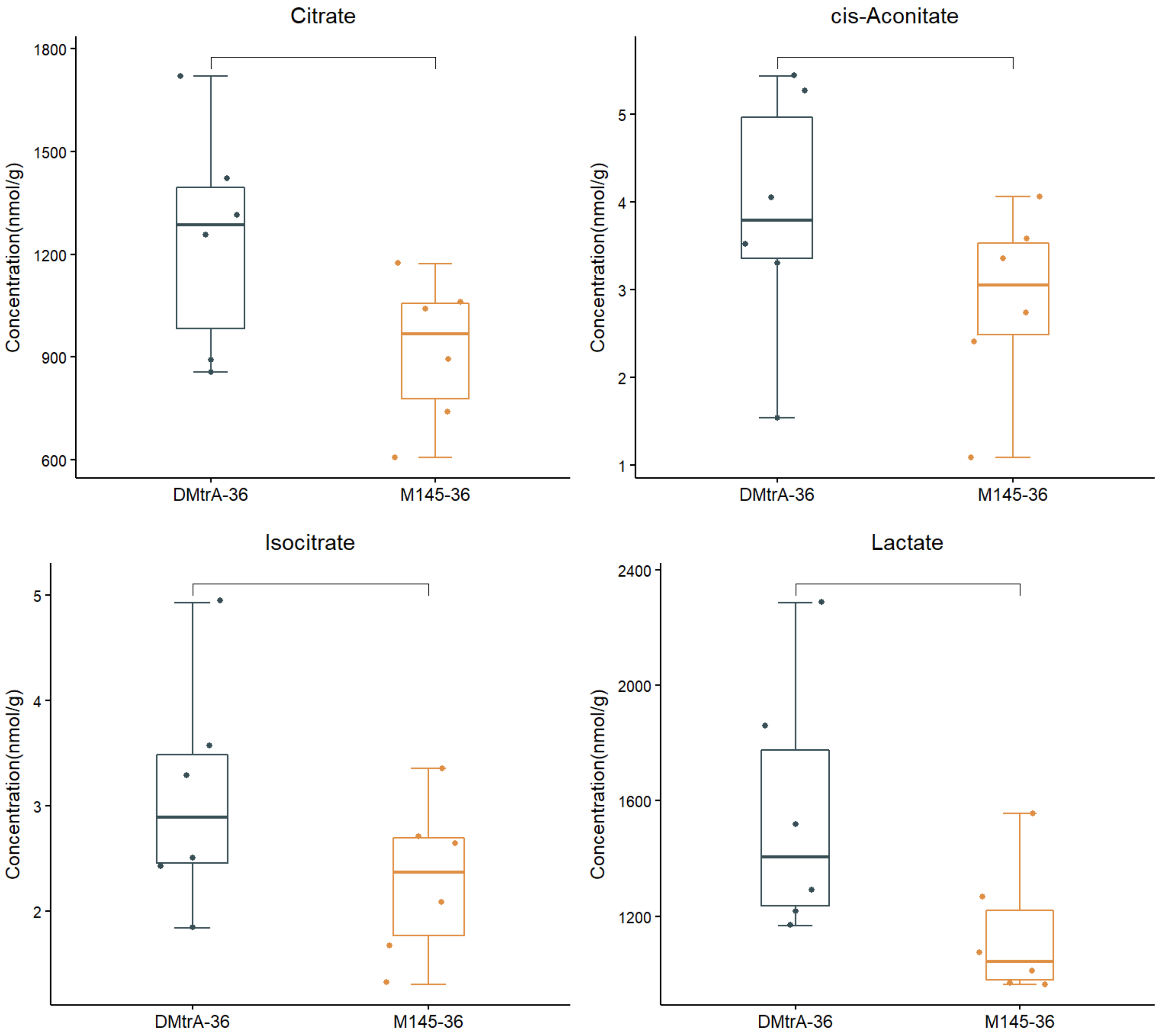

FIG S9 Quantification of TCA cycle metabolites on YBP at 36 h. *S. coelicolor* wild-type strain M145 and Δ*mtrA* were grown at 30° C on solid YBP medium for 36 h; the boxplot data were obtained using six different sample preparations. Student's t-test was used for comparison; ns, not significant.

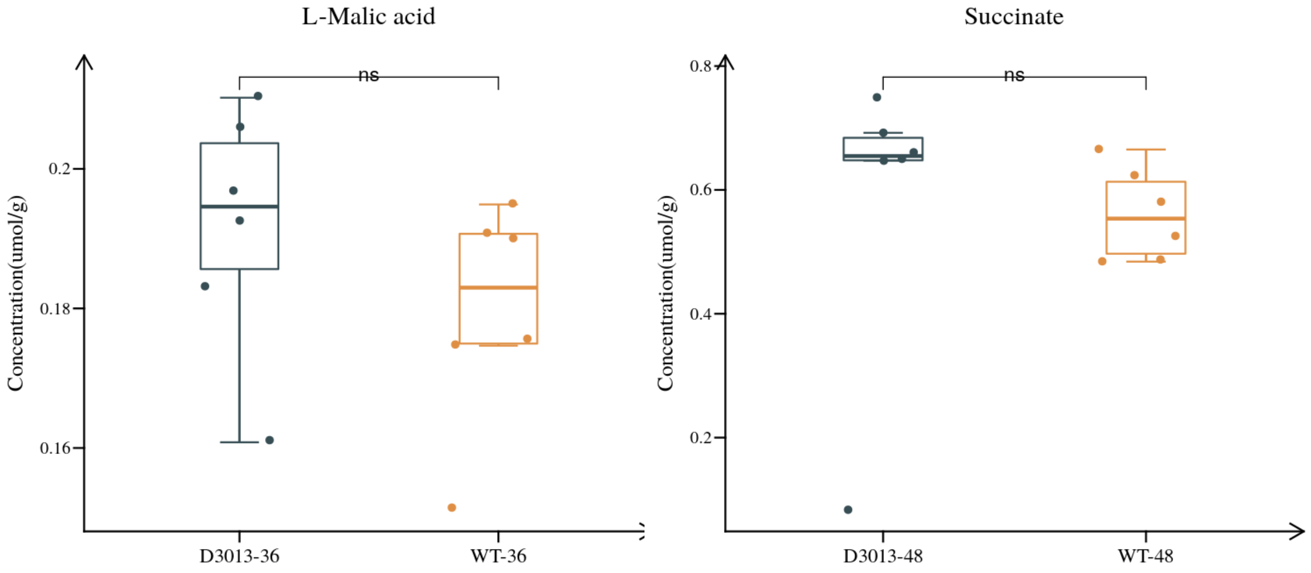

FIG S10 Quantification of the TCA cycle metabolites malic acid and succinate on R2YE. *S. coelicolor* wild-type strain M145 and Δ*mtrA* (D3013 is the *mtrA* mutant) were grown at 30° C on solid R2YE medium, and L-malic acid and succinate were measured at 36 h and 48 h, respectively. The boxplot data were obtained using six different sample preparations. Student's t-test was used for comparison; ns, not significant.

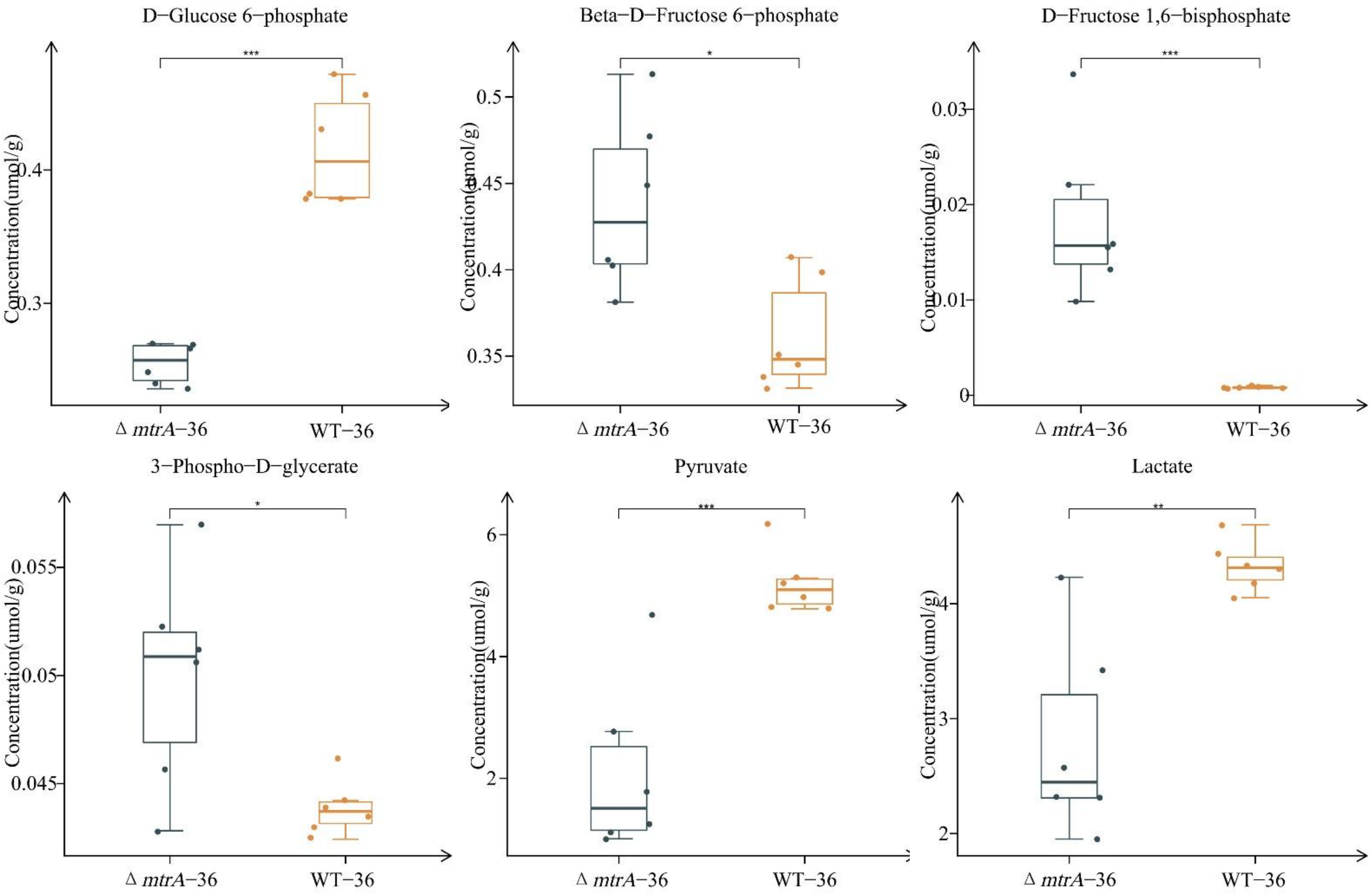

FIG S11 Quantification of glycolysis pathway metabolite production on R2YE at 36 h. *S. coelicolor* wild-type strain M145 and Δ*mtrA* were grown at 30° C on solid R2YE medium for 36 h; the boxplot data six different sample preparations. Student's t-test was used for comparison; *ρ<0.05, **ρ<0.01, ***ρ<0.005.

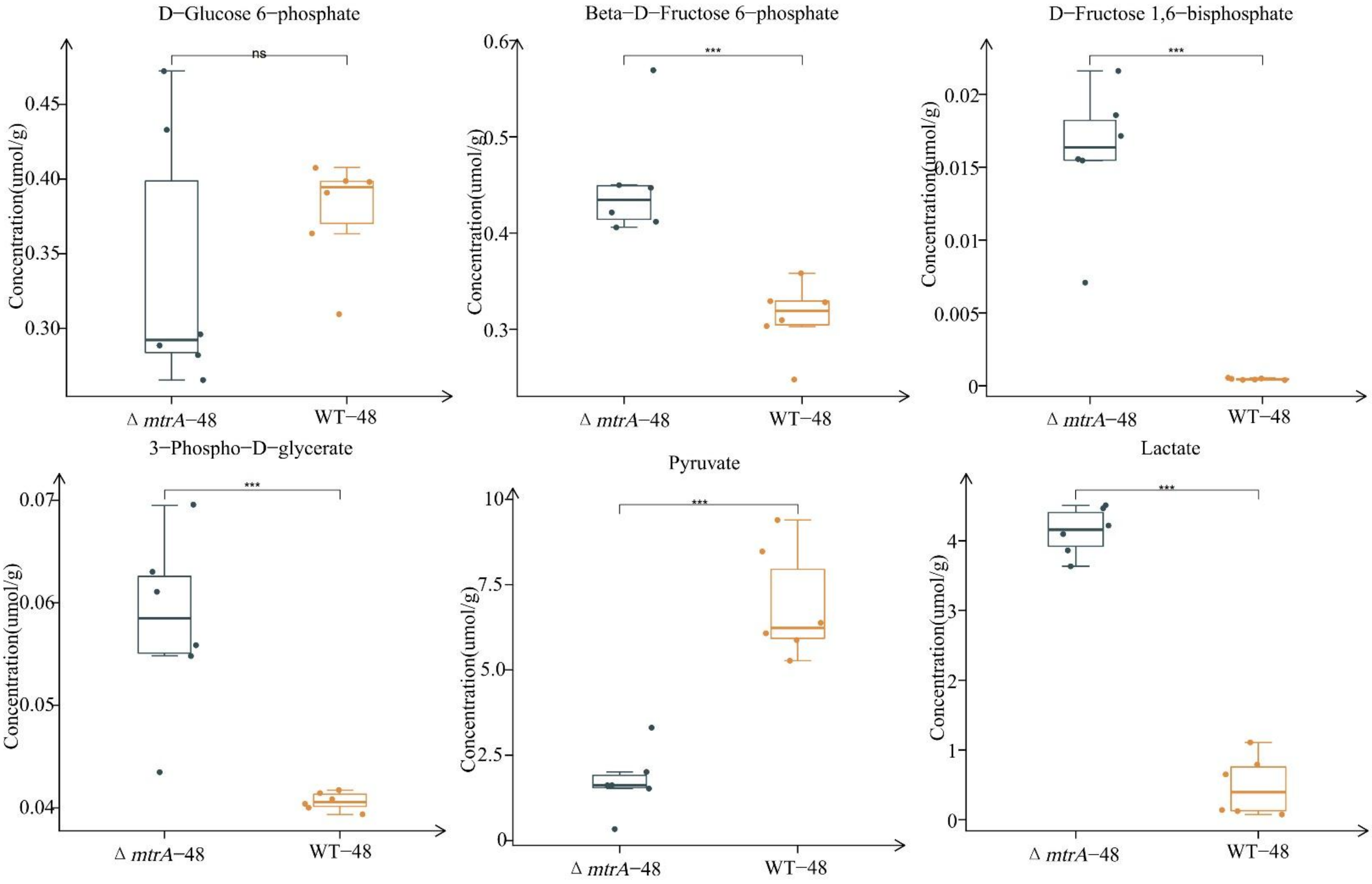

FIG S12 Quantification of glycolysis pathway metabolite production on R2YE at 48 h. *S. coelicolor* wild-type strain M145 and Δ*mtrA* were grown at 30° C on solid R2YE medium for 48 h; the boxplot data were obtained using six different sample preparations. Student's t-test was used for comparison; *ρ<0.05, **ρ<0.01, ***ρ<0.005.

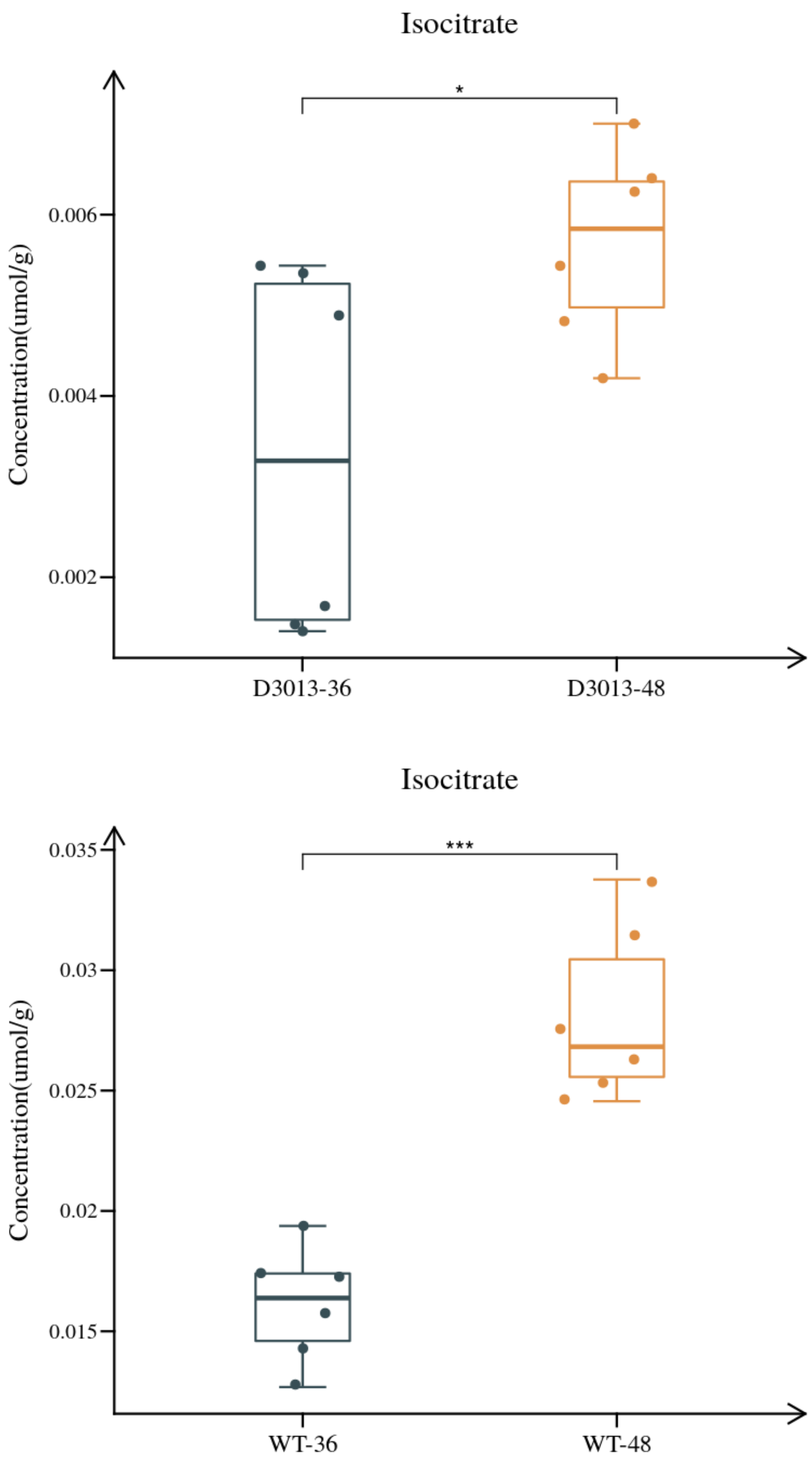

FIG S13 Dynamic analysis of metabolite production in M145 and Δ*mtrA*. *S. coelicolor* wild-type strain M145 and Δ*mtrA* were grown at 30° C on solid R2YE medium for 36 h or 48 h, and the indicated metabolites were measured. The boxplot data were obtained using six different sample preparations. Student's t-test was used for comparison; *ρ<0.05, **ρ<0.01, ***ρ<0.005.

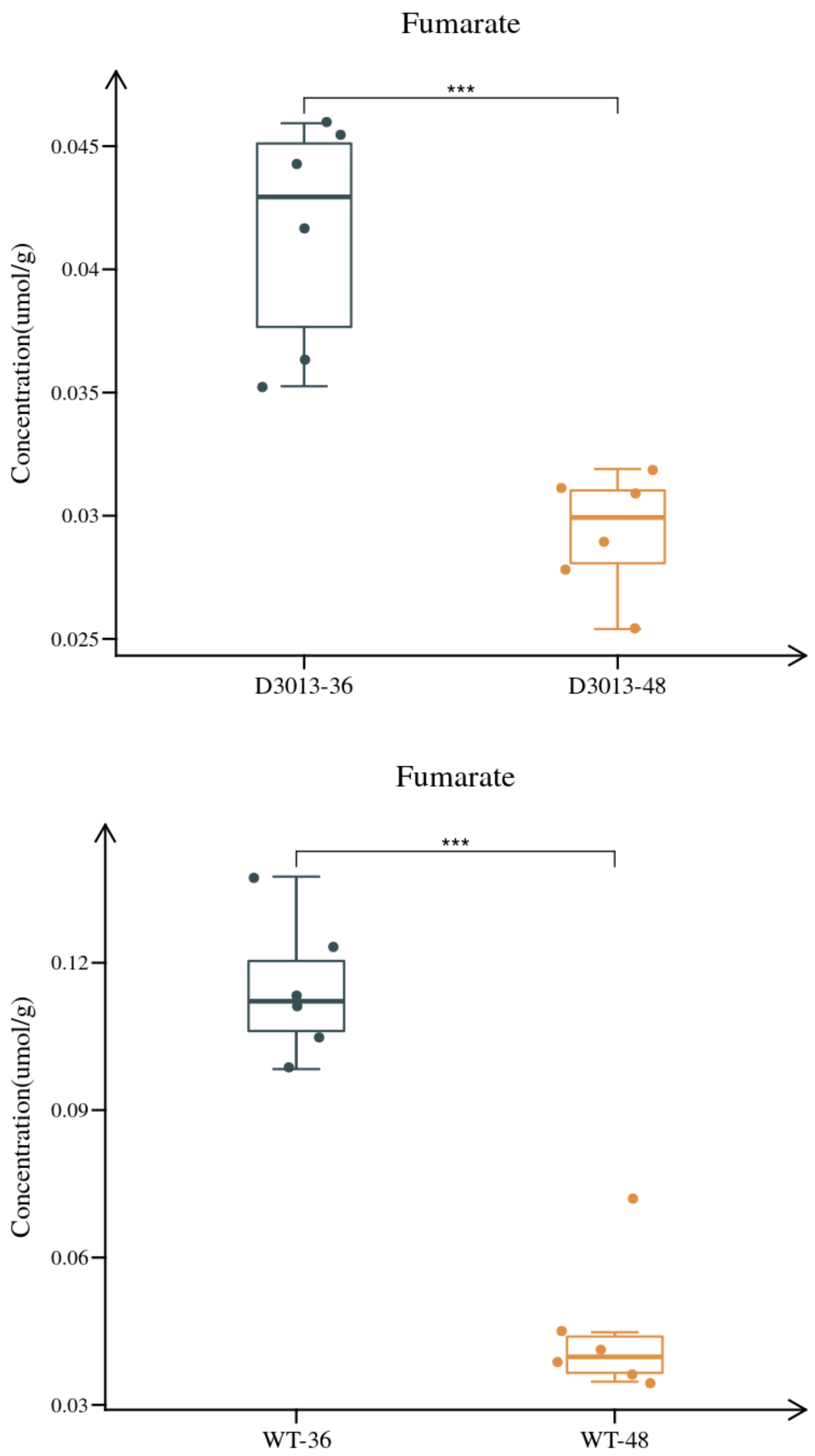

FIG S14 Dynamic analysis of metabolite production in M145 and Δ*mtrA*. *S. coelicolor* wild-type strain M145 and Δ*mtrA* were grown at 30° C on solid R2YE medium for 36 h or 48 h, and the indicated metabolites were measured. The boxplot data were obtained using six different sample preparations. Student's t-test was used for comparison; *ρ<0.05, **ρ<0.01, ***ρ<0.005.

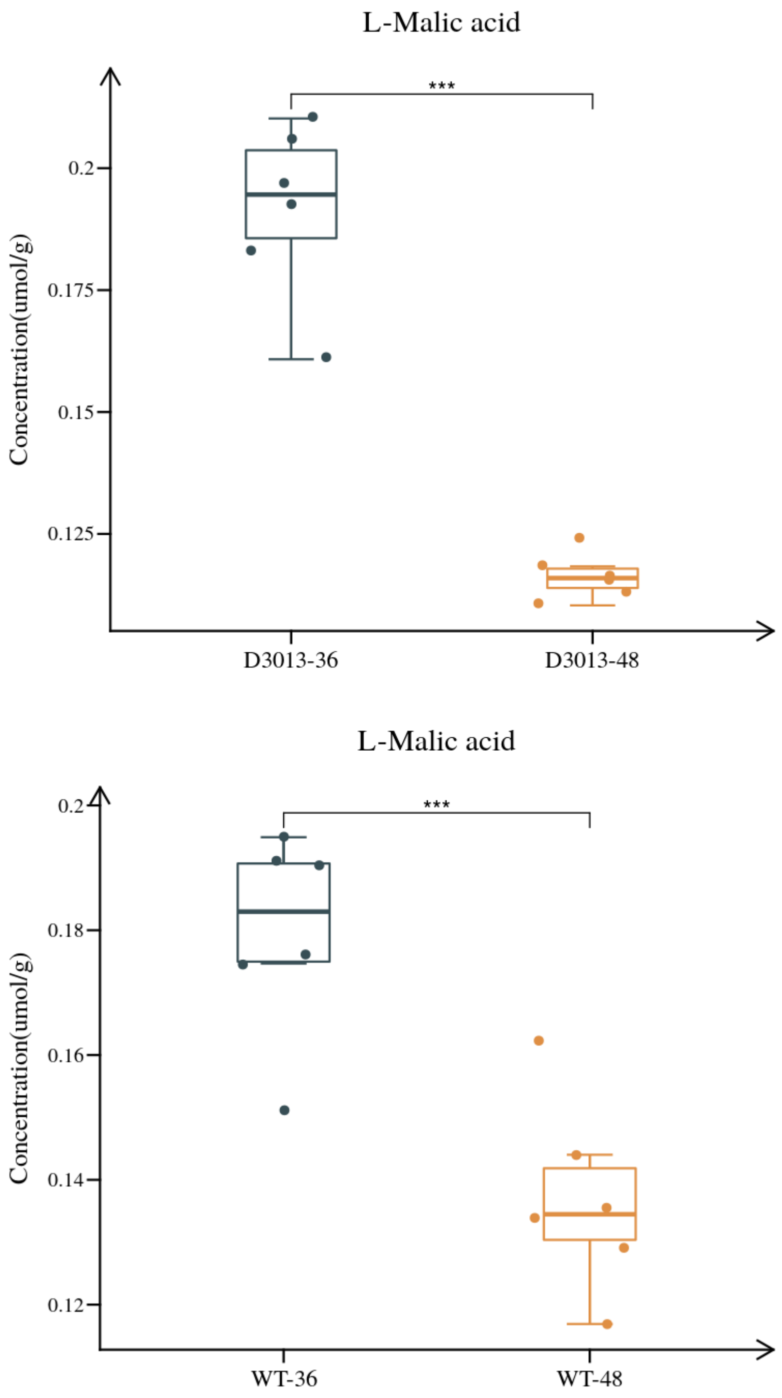

FIG S15 Dynamic analysis of metabolite production in M145 and Δ*mtrA*. *S. coelicolor* wild-type strain M145 and Δ*mtrA* were grown at 30° C on solid R2YE medium for 36 h or 48 h, and the indicated metabolites were measured. The boxplot data were obtained using six different sample preparations. Student's t-test was used for comparison; *ρ<0.05, **ρ<0.01, ***ρ<0.005.

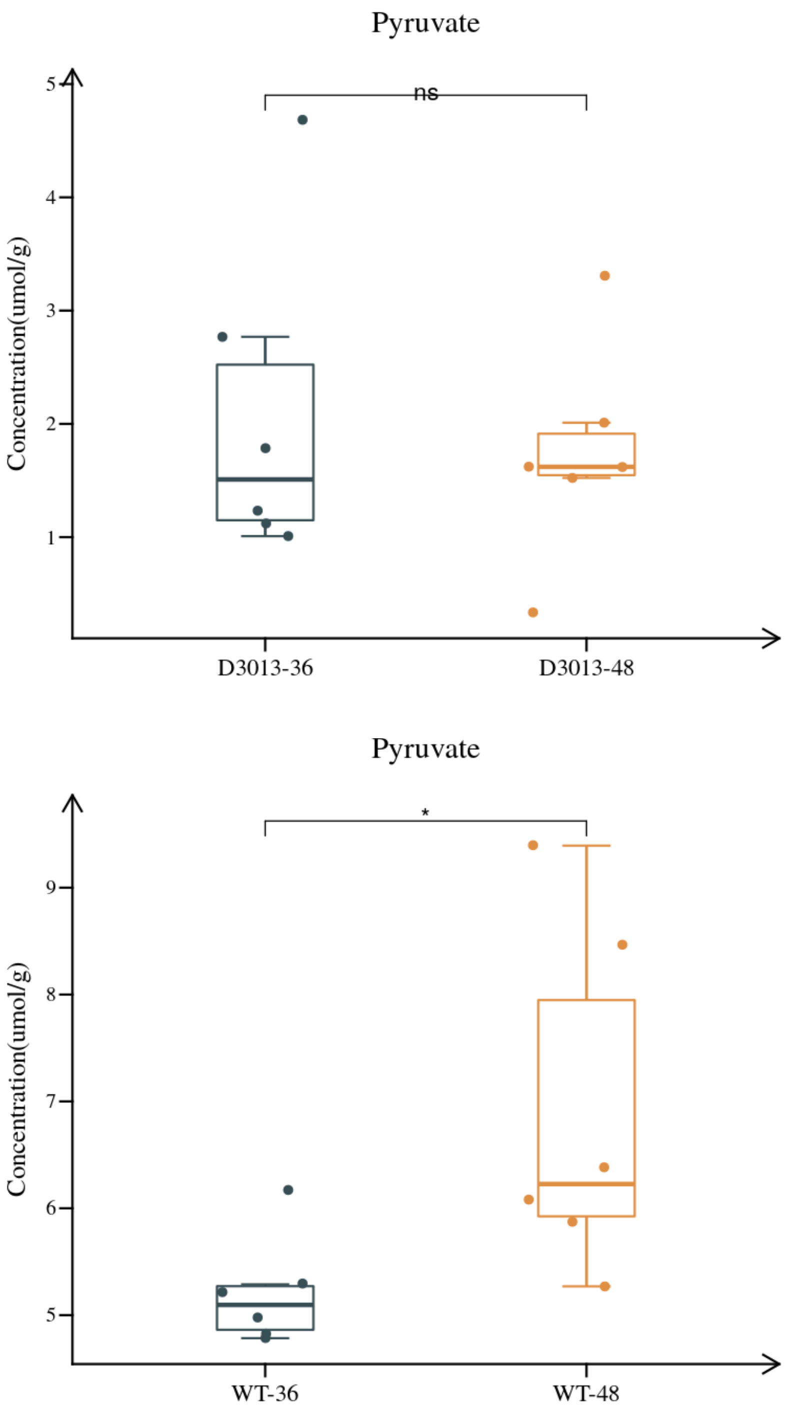

FIG S16 Dynamic analysis of metabolite production in M145 and Δ*mtrA*. *S. coelicolor* wild-type strain M145 and Δ*mtrA* were grown at 30° C on solid R2YE medium for 36 h or 48 h, and the indicated metabolites were measured. The boxplot data were obtained using six different sample preparations. Student's t-test was used for comparison; *$\rho<0.05$, **$\rho<0.01$, ***$\rho<0.005$.

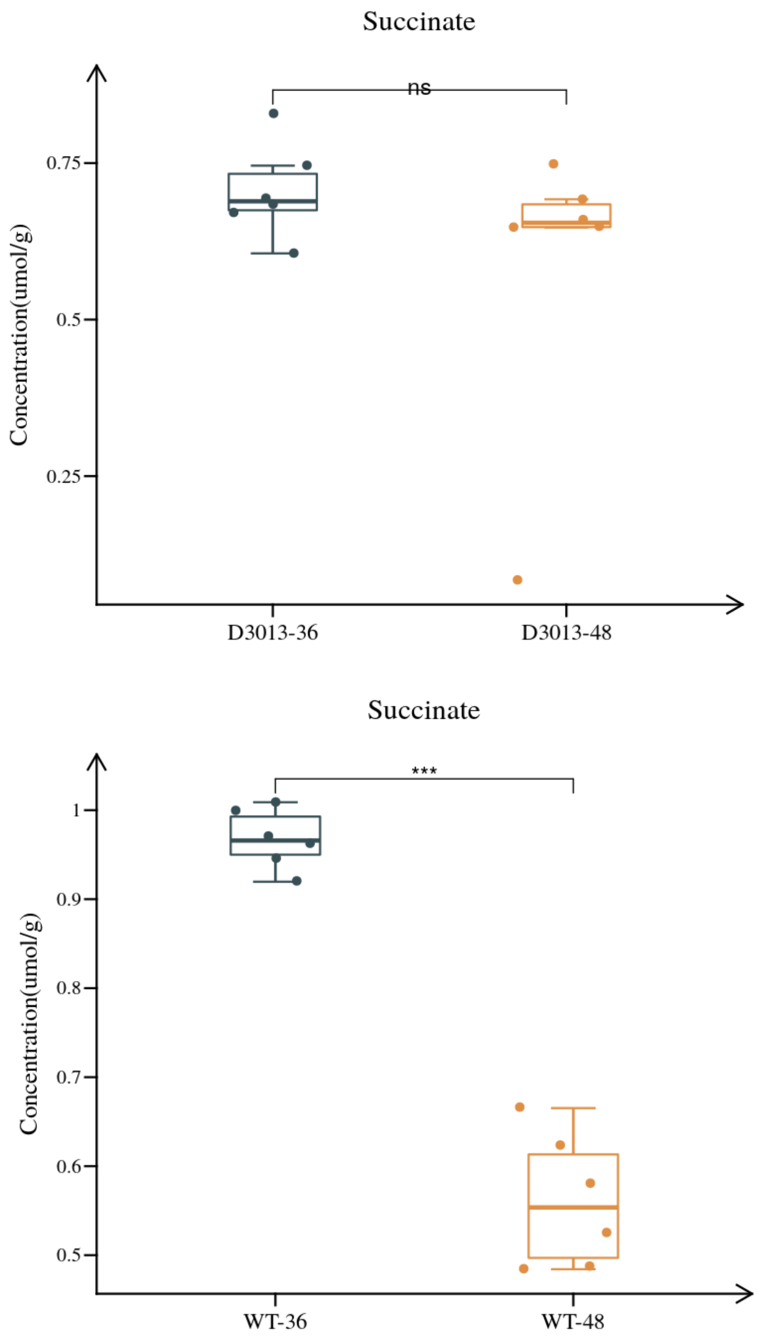

FIG S17 Dynamic analysis of metabolite production in M145 and Δ*mtrA*. *S. coelicolor* wild-type strain M145 and Δ*mtrA* were grown at 30° C on solid R2YE medium for 36 h or 48 h, and the indicated metabolites were measured. The boxplot data were obtained using six different sample preparations. Student's t-test was used for comparison; *ρ<0.05, **ρ<0.01, ***ρ<0.005.

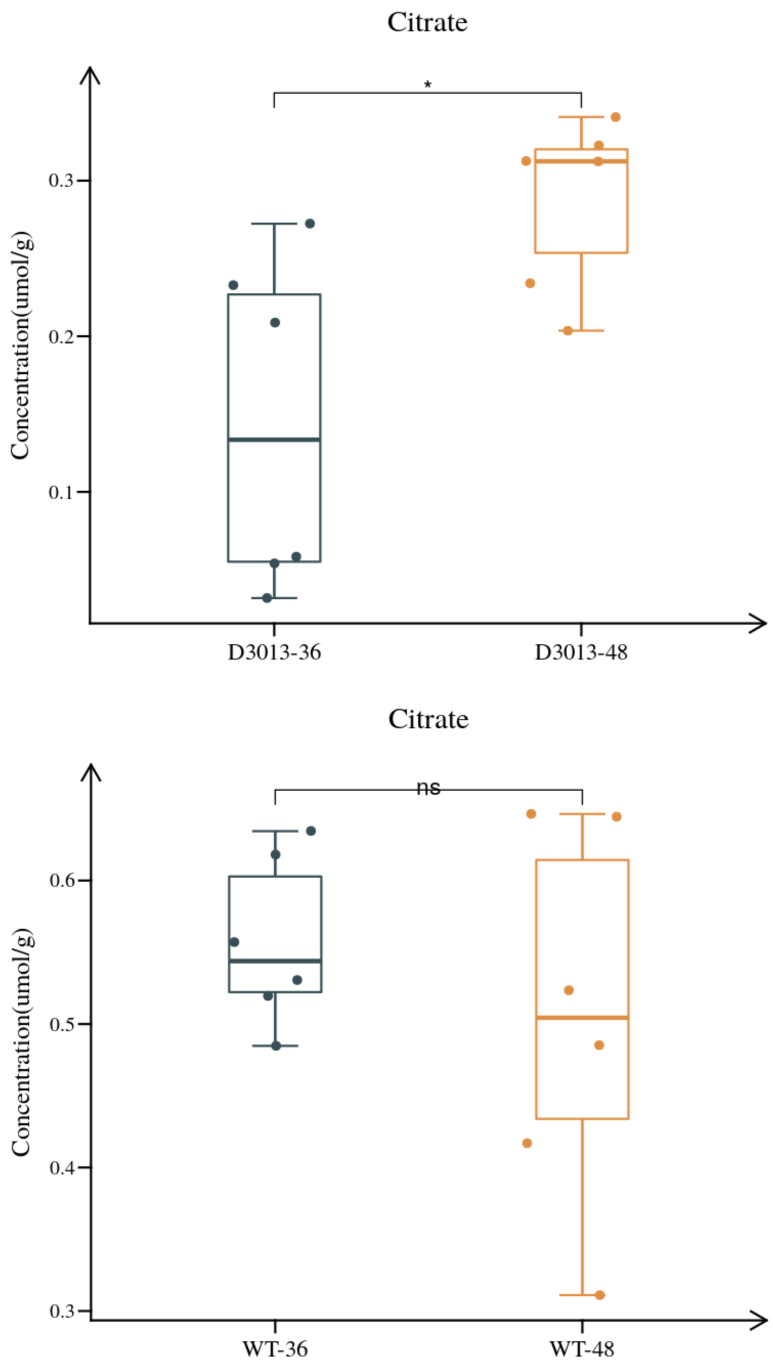

FIG S18 Dynamic analysis of metabolite production in M145 and Δ*mtrA*. *S. coelicolor* wild-type strain M145 and Δ*mtrA* were grown at 30° C on solid R2YE medium for 36 h or 48 h, and the indicated metabolites were measured. The boxplot data were obtained using six different sample preparations. Student's t-test was used for comparison; *$\rho<0.05$, **$\rho<0.01$, ***$\rho<0.005$.

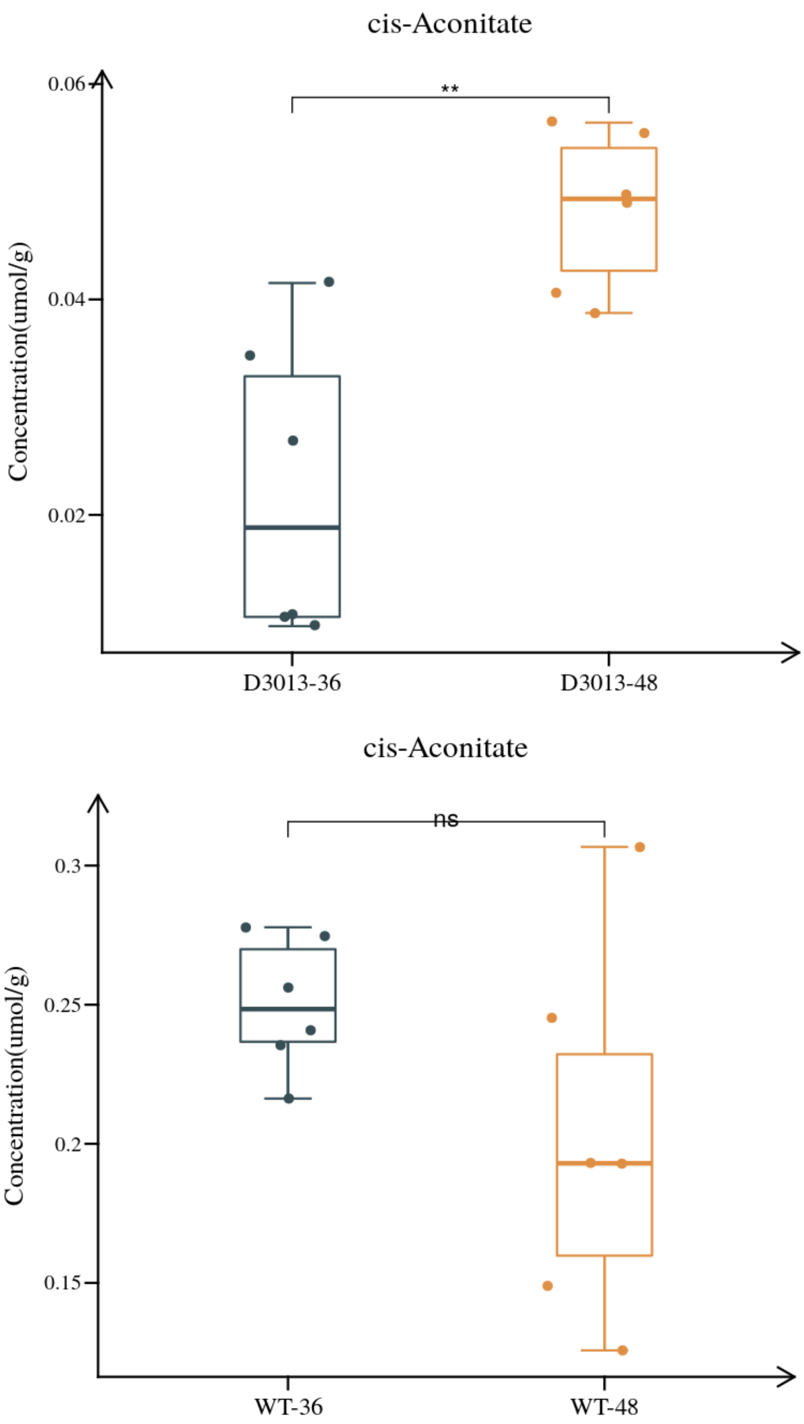

FIG S19 Dynamic analysis of metabolite production in M145 and Δ*mtrA*. *S. coelicolor* wild-type strain M145 and Δ*mtrA* were grown at 30° C on solid R2YE medium for 36 h or 48 h, and the indicated metabolites were measured. The boxplot data were obtained using six different sample preparations. Student's t-test was used for comparison; *ρ<0.05, **ρ<0.01, ***ρ<0.005.

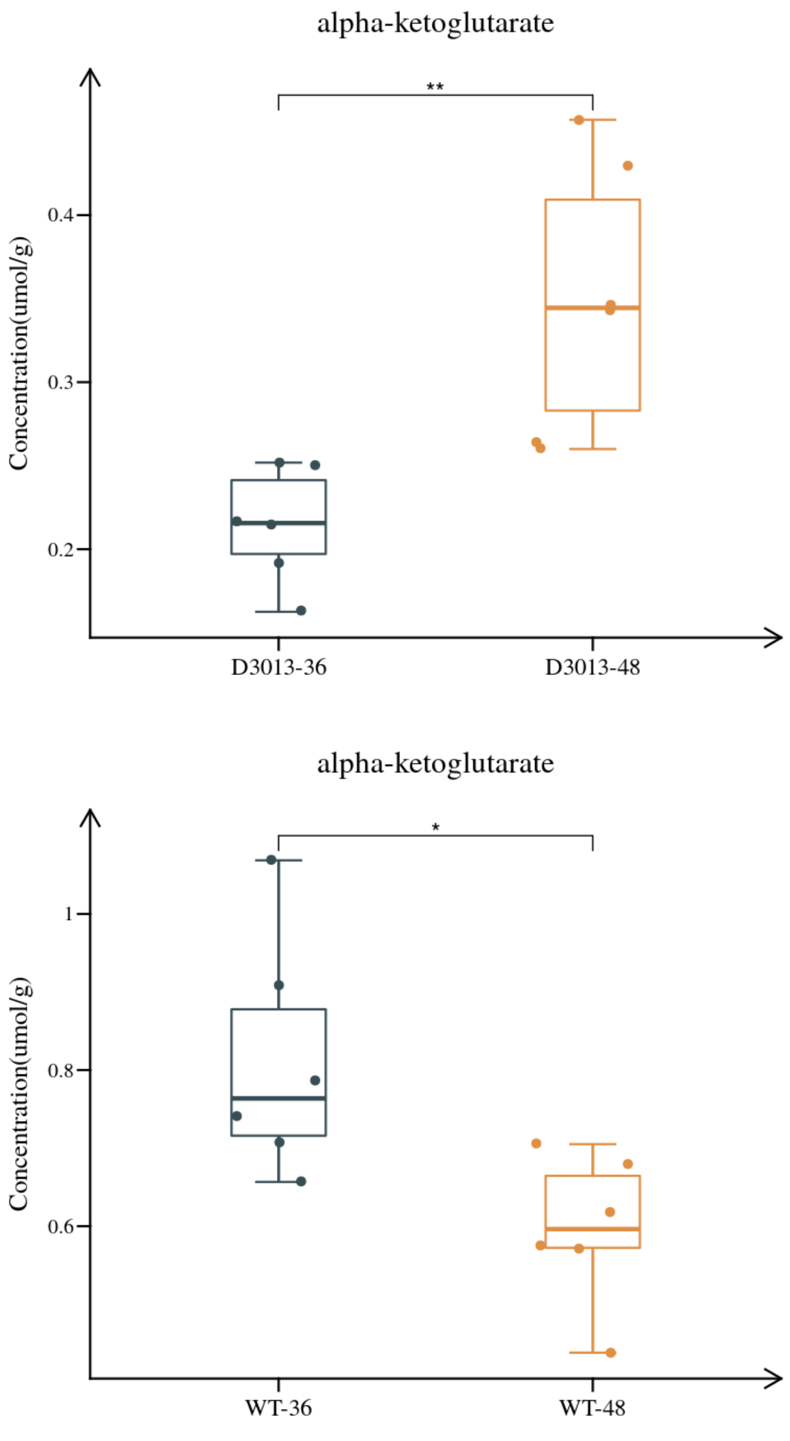

FIG S20 Dynamic analysis of metabolite production in M145 and Δ*mtrA*. *S. coelicolor* wild-type strain M145 and Δ*mtrA* were grown at 30° C on solid R2YE medium for 36 h or 48 h, and the indicated metabolites were measured. The boxplot data were obtained using six different sample preparations. Student's t-test was used for comparison; *ρ<0.05, **ρ<0.01, ***ρ<0.005.

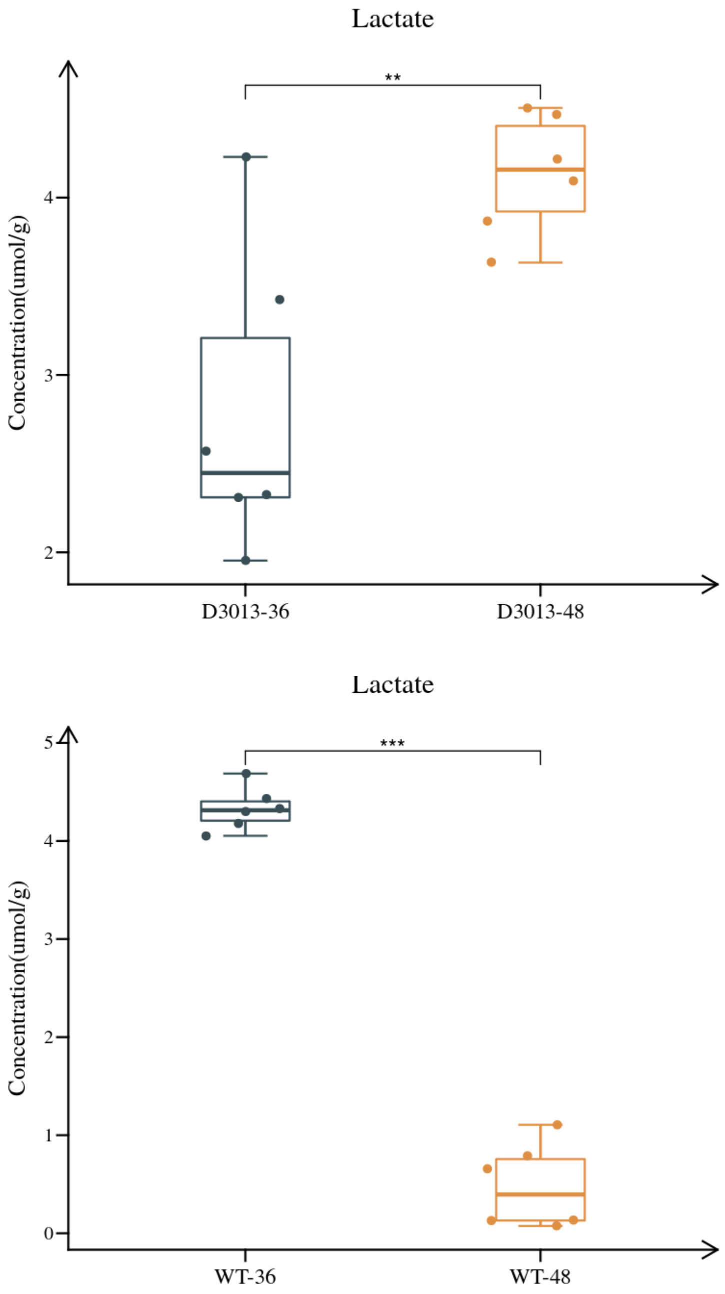

FIG S21 Dynamic analysis of metabolite production in M145 and Δ*mtrA*. *S. coelicolor* wild-type strain M145 and Δ*mtrA* were grown at 30° C on solid R2YE medium for 36 h or 48 h, and the indicated metabolites were measured. The boxplot data were obtained using six different sample preparations. Student's t-test was used for comparison; *ρ<0.05, **ρ<0.01, ***ρ<0.005.

Table S1. Potential MtrA sites upstream of carbon metabolism pathway genes in *S. lividans*

Gene	Function	Predicted MtrA site	Position to TSS
SLI_5128	putative succinate dehydrogenase iron-sulfur subunit	**GTCGC**GCGGCG**GTCAC**	-152
SLI_4879	2-oxoglutarate oxidoreductase, alpha subunit	**GTCAC**GGTGCG**CGGAC**	-71
SLI_3085	citrate synthase	**GTCAC**ACAGCA**CTTCC**	-160
SLI_4622	citrate synthase	**GTCAC**GCCGCC**CATCC**	-277
SLI_6099	citrate synthase	**TTGAC**TTAACT**GTCCA**	-92
SLI_6100	citrate synthase		-16
SLI_4068-bkdA1	putative branched-chain alpha keto acid dehydrogenase E1 alpha subunit	**GCGAA**CAGCAC**GTTAC**	-175
SLI_4069	putative two-component system response transcriptional regulator		-132
SLI_4081-bkdC2	putative dihydrolipoamide acyltransferase component E2	**GTGAC**GCTCCA**GCATC**	-65
SLI_4082	putative branched-chain alpha keto acid dehydrogenase E1 beta subunit	**GCGCG**GAGCTG**GTCCC**	-63
SLI_4083	E1-alpha branched-chain alpha keto acid dehydrogenase	**GTCAC**ACCCGT**GGCGA**	-33
SLI_4084	putative transcriptional regulator		-141

The potential MtrA site was predicted using MEME software; TSS, translational start site.

Table S2. Potential MtrA sites upstream of carbon metabolism pathway genes in *S. venezuelae*

Gene	Function	Predicted MtrA site	Position to TSS
SVEN_4531	putative succinate dehydrogenase iron-sulfur subunit	**GTCCC**GCGGCG**GTCAC**	-152
SVEN_4304	2-oxoglutarate oxidoreductase, alpha subunit	**GTCCC**CTTGCC**GCGAG**	-14
SVEN_2535	citrate synthase	**GAGAC**AGTCAG**GGCAC**	-35
SVEN_4205	citrate synthase	**GTGAC**ATCCGG**CACAC**	-106
SVEN_3587 *bkdA1*	putative branched-chain alpha keto acid dehydrogenase E1 alpha subunit	**GTCAT**AGGCAC**GTTAC**	-133
SVEN_3588	putative two-component system response transcriptional regulator		-189
SVEN_3603	putative dihydrolipoamide acyltransferase component E2	**GTCGC**GCGACT**GCAGT**	-16
SVEN_3604	putative branched-chain alpha keto acid dehydrogenase E1 beta subunit	**GTGCC**GAGCTC**GAAGC**	-15
SVEN_3605	E1-alpha branched-chain alpha keto acid dehydrogenase	**GAGGC**ATTTTG**GCTAC**	-80
SVEN_3606	putative transcriptional regulator		-177

The potential MtrA site was predicted using MEME software; TSS, translational start site.

Table S3. Potential MtrA sites upstream of carbon metabolism pathway genes in *S. avermitilis*

Gene	Function	Predicted MtrA site	Position to TSS
SAV_3398-sdhB1	putative succinate dehydrogenase iron-sulfur subunit	**GTCCC**GCGGCG**GTCAC**	-152
SAV_4877-korA	2-oxoglutarate oxidoreductase, alpha subunit	**GTGAC**CAGCCA**GGTCA**<br>**GTCAC**TGAACT**GGACC**	16<br>5
SAV_5330-citA1	citrate synthase	**GAGAC**AGTCAG**GGCAC**	-35
SAV_3859-citA2	citrate synthase	**GTGAG**ATCGAG**GTGAC**	-162
SAV_2428	citrate synthase	**TTGAT**TCGACT**GTCCA**	-65
SAV_2427-citA3	citrate synthase		-15
SAV_4376-bkdF	putative branched-chain alpha keto acid dehydrogenase E1 alpha subunit	**GCACC**ATGCAC**GTTAC**	-165
SAV_4375	putative two-component system response transcriptional regulator		-233
SAV_4364-bkdC	putative dihydrolipoamide acyltransferase component E2	**GCCTC**CAGATG**GTGGA**	-126
SAV_4363-bkdB	putative branched-chain alpha keto acid dehydrogenase E1 beta subunit	**TTGAC**CATCGG**GCCCC**	-51
SAV_4362-bkdA	E1-alpha branched-chain alpha keto acid dehydrogenase	**GAACG**CACTAC**GTCCA**	-135
SAV_4361	AsnC-family transcriptional regulator		-38

The potential MtrA site was predicted using MEME software; TSS, translational start site.

Table S4. Potential MtrA sites upstream of carbon metabolism pathway genes in *Mycobacterium tuberculosis*

Gene	Function	Predicted MtrA site	Position to TSS
Rv0889c<br>*gltA*	citrate synthase	**GTCAC**GAGGGT**GAGCC**<br>**GTGCC**GCCTCG**TCTAC**<br>**GTTCG**GTCGGG**GTCAA**<br>**GTCAG**CGAGGG**GTTAT**	-85<br>-106<br>-259<br>-226
Rv0896<br>*gltA2*	citrate synthase	**GTTAA**ACGGAC**GCCGC**<br>**GTGCC**AGAGAC**CCCAC**<br>**GTGTC**GGCCAC**GAGAA**<br>**CTACA**GGTGAT**GTTCA**	-161<br>-91<br>-5<br>-335
Rv1131<br>*prpC*	methylcitrate synthase PrpC	**GTTCA**ACGGGT**TCCAC**<br>**TCGAC**CAACAC**GTTCA**<br>**GGATC**GGCGCA**GTGAT**	-127<br>-48<br>-300
Rv2454c	oxidoreductase (beta subunit)	**GTTAC**CCCAAG**GTGGT**<br>**GTGGC**TCCGGA**GTTGA**<br>**GGGAA**CGGGTT**GAGAT**	-219<br>-205<br>-261
Rv2455c	oxidoreductase (alpha subunit)	**GTTAT**CGGACA**GGCAC**<br>**GCACG**GCTTGT**GTCAC**	-17<br>-83
Rv3319<br>*sdhB*	succinate dehydrogenase (iron-sulphur protein subunit) SdhB (succinic dehydrogenase) (fumarate reductase) (fumarate dehydrogenase) (fumaric hydrogenase)	**GTCCC**GCGGCG**GTCAC**<br>**GTCAA**CTACAT**GCGAC**	-167<br>-121
Rv2495c<br>*bkdC*	putative branched-chain keto acid dehydrogenase E2 component BkdC	**GTTAC**CCGGCC**CCGAC**<br>**GTGCC**GGTCTG**GCCGC**	-38<br>-153
Rv2496c<br>*bkdB*	putative branched-chain keto acid dehydrogenase E1 component, beta subunit BkdB	**GTGAC**GGCTCG**GGCAA**<br>**GTAGC**TCGGAC**CGCAC**	-159<br>-138
Rv2497c<br>*bkdA*	putative branched-chain keto acid dehydrogenase E1 component, alpha subunit BkdA	**GTAAC**CGCGTT**GTGAT**<br>**GAAGC**GCCAGT**GTTGC**	-19<br>-164

The potential MtrA site was predicted using MEME software; TSS, translational start site.

Table S5. Potential MtrA sites upstream of carbon metabolism pathway genes in *Amycolatopsis mediterranei*

Gene	Function	Predicted MtrA site	Position to TSS
AMED_4853 gltA	citrate synthase	**GTCAG**CGCGTC**GAACC**	-227
AMED_8426 gltA	citrate synthase	**GTCAC**CGCTGC**GCGAA**	-150
AMED_8451 gltA	citrate synthase	**GTGAC**TGAATC**GTTCT**	-166
AMED_0540 korB	2-oxoglutarate/2-oxoacid ferredoxin oxidoreductase subunit beta	**GTGCC**GGAGAT**GAACC**	-145
AMED_0541 korA	2-oxoglutarate/2-oxoacid ferredoxin oxidoreductase subunit alpha	**GTCAG**ATACCT**GTAAC**	-172
AMED_0849 sdhB	succinate dehydrogenase iron-sulfur protein	**GTGGC**CGCCGC**GGGAC**	-149
AMED_0852 sdhC	succinate dehydrogenase cytochrome b-556 subunit	**GTGAT**GGACCA**GACAC**	-160
AMED_2352 sdhA	succinate dehydrogenase flavoprotein subunit	**GGAAT**CCTTTT**ATGAC**	-11
AMED_3219 sdhC	succinate dehydrogenase cytochrome b-556 subunit	**TTGAC**CTCTGG**TCGAT**	-20
AMED_7570 pdhB	pyruvate dehydrogenase E1 component subunit beta	**GTTAC**CGCGAC**GCCGA**	-249
AMED_7571 pdhA	pyruvate dehydrogenase E1 component subunit alpha	**TGGAC**GTTTGG**GTGCC**	-110

The potential MtrA site was predicted using MEME software; TSS, translational start site.

Table S6. Potential MtrA sites upstream of carbon metabolism pathway genes in *Corynebacterium glutamicum*

Gene	Function	Predicted MtrA site	Position to TSS
NCgl0795<br>*Cgl0829*<br>*gltA*	citrate synthase	**GTCAC**GCCAAT**AGAAC**	-242
NCgl1482<br>*Cgl1540*	aconitate hydratase	**GTGAC**TTTCTA**TCACC**<br>**GTTCT**TGGCGC**GTCAA**	-79<br>-184
NCgl0359<br>*Cgl0370*	succinate dehydrogenase cytochrome b subunit	**GTCAC**GGGGCG**CTCCC**<br>**GTGAC**TGGTTA**ATGCC**<br>**GTGCC**ATGACT**GTTAG**<br>**GTGAC**ACAATT**ATCCC**	-51<br>-40<br>-5<br>-315
NCgl0360<br>*Cgl0371*	succinate dehydrogenase/fumarate reductase, flavoprotein subunits	**GTTGC**CGATCA**GGACC**<br>**GGTAC**ATCATT**GCCAA**	-58<br>-91
NCgl0361<br>*Cgl0372*	succinate dehydrogenase/fumarate reductase Fe-S protein	**GTCCC**ACTGCA**GACAA**	-13
NCgl0355<br>*Cgl0366*<br>*lpdA*	dihydrolipoamide dehydrogenase/glutathione oxidoreductase and related enzymes	**GTTCT**TTTGTA**GTCCC**<br>**GTTCA**TGAATT**GGCAT**	-352<br>-285
NCgl2167<br>*aceE*	pyruvate dehydrogenase (acetyl-transferring)	**GTCAC**AATTAG**GTACG**<br>**GTGAC**GTAAGG**GGAGC**	-67<br>-160

The potential MtrA site was predicted using MEME software; TSS, translational start site.

Table S7. Potential MtrA sites upstream of carbon metabolism pathway genes in *Mycobacterium smegmatis*

Gene	Function	Predicted MtrA site	Position to TSS
MSMEG_4035-gltA	citrate synthase	**GCGGA**AACTCC**GTCAC**<br>**GTAAG**GAGGCA**GCTTC**	-99<br>-139
MSMEG_5672-gltA	citrate synthase I	**GTGAC**CGTCGA**GCGCA**<br>**GTGAG**CAGTTG**GTCAC**	-189<br>-106
MSMEG_5676	citrate synthase	**GTATC**CCCACC**GCGAC**<br>**ACACC**GAGCGC**GTCAC**	-35<br>-278
MSMEG_6647	citrate synthase II	**GTCAC**CACGCT**GCGGC**	-172
MSMEG_4645-orB	alpha oxoglutarate ferredoxin oxidoreductase, beta subunit	**GTGCC**CGAGAT**GAACC**<br>**GTCAT**GCGTTC**GCTCC**	-226<br>-1
MSMEG_4646	pyruvate synthase	**GTCAC**CCACGG**CTCCC**<br>**GCTAC**CCATCG**GTAGT**	-7<br>-283
MSMEG_1672-sdhC	succinate dehydrogenase, cytochrome b556 subunit	**GGCGC**ATATGC**GTGCC**<br>**GTTAG**GCTTAC**CTATC**	-272<br>-137
MSMEG_0417	succinate dehydrogenase	**GCTAC**GCCAAT**GTGAC**<br>**CCGAC**GTGACG**GTGAC**	-341<br>-116
MSMEG_0418	succinate dehydrogenase flavoprotein subunit	**GTCAG**CCAGGC**GGATC**<br>**GTGCC**GAGGGT**GATCC**	-44<br>-155
MSMEG_1669-sdhB	succinate dehydrogenase, iron-sulfur protein	**GTGCC**CTGCTT**GTAGG**	-81
MSMEG_2471	pyruvate dehydrogenase alpha subunit	**GTCAC**CCAGCG**GAAAC**	-304
MSMEG_4323-aceE	pyruvate dehydrogenase E1 component	**GTCGC**CCCAAA**CTGCC**	-16
MSMEG_4710	dihydrolipoamide acetyltransferase	**GAGGC**TCCCGT**GTTGC**	-106
MSMEG_4711-pdhB	pyruvate dehydrogenase E1 component subunit beta	**GTGCA**CCCGAC**GTCGA**	-98
MSMEG_4712-pdhA	pyruvate dehydrogenase E1 component, alpha subunit	**GTGGC**CGTCCG**CTGGC**<br>**GCCTC**CTTCAG**GTGAC**	-167<br>-3

The potential MtrA site was predicted using MEME software; TSS, translational start site.
